# The Histogram Analysis of Intravoxel Incoherent Motion-Kurtosis Model in the Diagnosis and Grading of Prostate Cancer—A Preliminary Study

**DOI:** 10.3389/fonc.2021.604428

**Published:** 2021-10-27

**Authors:** Chunmei Li, Lu Yu, Yuwei Jiang, Yadong Cui, Ying Liu, Kaining Shi, Huimin Hou, Ming Liu, Wei Zhang, Jintao Zhang, Chen Zhang, Min Chen

**Affiliations:** ^1^Department of Radiology, Beijing Hospital, National Center of Gerontology, Institute of Geriatric Medicine, Chinese Academy of Medical Sciences, Beijing, China; ^2^Philips Healthcare, Shanghai, China; ^3^Department of Urology, Beijing Hospital, National Center of Gerontology, Institute of Geriatric Medicine, Chinese Academy of Medical Sciences, Beijing, China; ^4^Department of Pathology, Beijing Hospital, National Center of Gerontology, Institute of Geriatric Medicine, Chinese Academy of Medical Sciences, Beijing, China

**Keywords:** prostate cancer, intravoxel incoherent motion, kurtosis, monoexponential, histogram analysis

## Abstract

**Objectives:**

This study was conducted in order to explore the value of histogram analysis of the intravoxel incoherent motion-kurtosis (IVIM-kurtosis) model in the diagnosis and grading of prostate cancer (PCa), compared with monoexponential model (MEM).

**Materials and Methods:**

Thirty patients were included in this study. Single-shot echo-planar imaging (SS-EPI) diffusion-weighted images (*b*-values of 0, 20, 50, 100, 200, 500, 1,000, 1,500, 2,000 s/mm^2^) were acquired. The pathologies were confirmed by in-bore MR-guided biopsy. The postprocessing and measurements were processed using the software tool Matlab R2015b for the IVIM-kurtosis model and MEM. Regions of interest (ROIs) were drawn manually. Mean values of *D*, D*, *f*, *K*, ADC, and their histogram parameters were acquired. The values of these parameters in PCa and benign prostatic hyperplasia (BPH)/prostatitis were compared. Receiver operating characteristic (ROC) curves were used to investigate the diagnostic efficiency. The Spearman test was used to evaluate the correlation of these parameters and Gleason scores (GS) of PCa.

**Results:**

For the IVIM-kurtosis model, *D* (mean, 10th, 25th, 50th, 75th, 90th), D* (90th), and *f* (10th) were significantly lower in PCa than in BPH/prostatitis, while *D* (skewness), D* (kurtosis), and *K* (mean, 75th, 90th) were significantly higher in PCa than in BPH/prostatitis. For MEM, ADC (mean, 10th, 25th, 50th, 75th, 90th) was significantly lower in PCa than in BPH/prostatitis. The area under the ROC curve (AUC) of the IVIM-kurtosis model was higher than MEM, without significant differences (*z* = 1.761, *P* = 0.0783). *D* (mean, 50th, 75th, 90th), D* (mean, 10th, 25th, 50th, 75th), and *f* (skewness, kurtosis) correlated negatively with GS, while *D* (kurtosis), D* (skewness, kurtosis), *f* (mean, 75th, 90th), and *K* (mean, 75th, 90th) correlated positively with GS. The histogram parameters of ADC did not show correlations with GS.

**Conclusion:**

The IVIM-kurtosis model has potential value in the differential diagnosis of PCa and BPH/prostatitis. IVIM-kurtosis histogram analysis may provide more information in the grading of PCa than MEM.

## Introduction

Prostate cancer (PCa) is the most common malignancy and the leading cause of cancer-related deaths among the elderly male (1). The accurate diagnosis and grading is critical in the appropriate treatment strategy and prognosis of PCa patients. Multiparametric MRI plays an important role in the detection, characterization, and staging of prostate cancer. The second edition of the Prostate Imaging Reporting and Data System version 2 (PI-RADS v2) regarded diffusion-weighted imaging (DWI) as one of the two dominant sequences to evaluate PCa (2). The monoexponential model (MEM), universally used in the clinic, is based on Gaussian distribution of tissue. However, non-Gaussian diffusion and blood microcirculation could both affect the characterization of PCa. Nowadays, DWI combined with various mathematical models has been gaining substantial interest as a possible tool to detect PCa, including intravoxel incoherent motion (IVIM) and kurtosis models.

The IVIM model was introduced by Le Bihan et al. (3), which uses the biexponential model and can simultaneously assess diffusion and perfusion. Despite the controversy of the advantages of the IVIM model, some studies have demonstrated that parameters derived from IVIM could improve the sensitivity and specificity of PCa diagnosis, as well as the stratification of PCa (4, 5). Diffusion kurtosis imaging (DKI) model can provide information about non-Gaussian diffusion and reflect microstructural complexity of tumor tissues (6). Previous studies reported that the DKI model was useful in the detection and assessment of PCa aggressiveness and may even be superior to MEM (7–10). Recently, a combination of IVIM and kurtosis (IVIM-kurtosis) model was introduced (11), which could add extra microstructural information regarding molecular diffusion, perfusion, and non-Gaussian information simultaneously. It was reported that the IVIM-kurtosis model had potential value for the diagnosis and prediction of distant metastasis of tumor (12, 13). However, no previous reports focused on the application of the IVIM-kurtosis model in PCa.

Heterogeneity is an important characteristic of tumors (14, 15). It exists at the cell level and is highly affected by the genetic background and origin as well as the environment where it establishes (16). Understanding heterogeneity is helpful for diagnosis, treatment, and prognosis. The histogram analysis is the most popular method for characterization of tumor heterogeneity and provides comprehensive information compared with the mean value (16, 17). It has been used in various DWI models for PCa, such as MEM, IVIM, and DKI models. Previous studies found that histogram analysis had better repeatability than traditional mean value measurement (18), improved diagnosis accuracy for PCa (19–21), and provided better imaging biomarker for aggressiveness evaluation for PCa than mean value (22). Though the histogram analysis has been widely used in in PCa, especially for MEM, the value of histogram analysis for the IVIM-kurtosis model in PCa is still unknown.

In this study, we sought to explore the value of histogram analysis for the IVIM-kurtosis model in the diagnosis and grading of PCa, and compare with MEM, with in-bore transrectal MR-guided biopsy as pathological reference.

## Materials and Methods

### Participants

**Figure 1:**
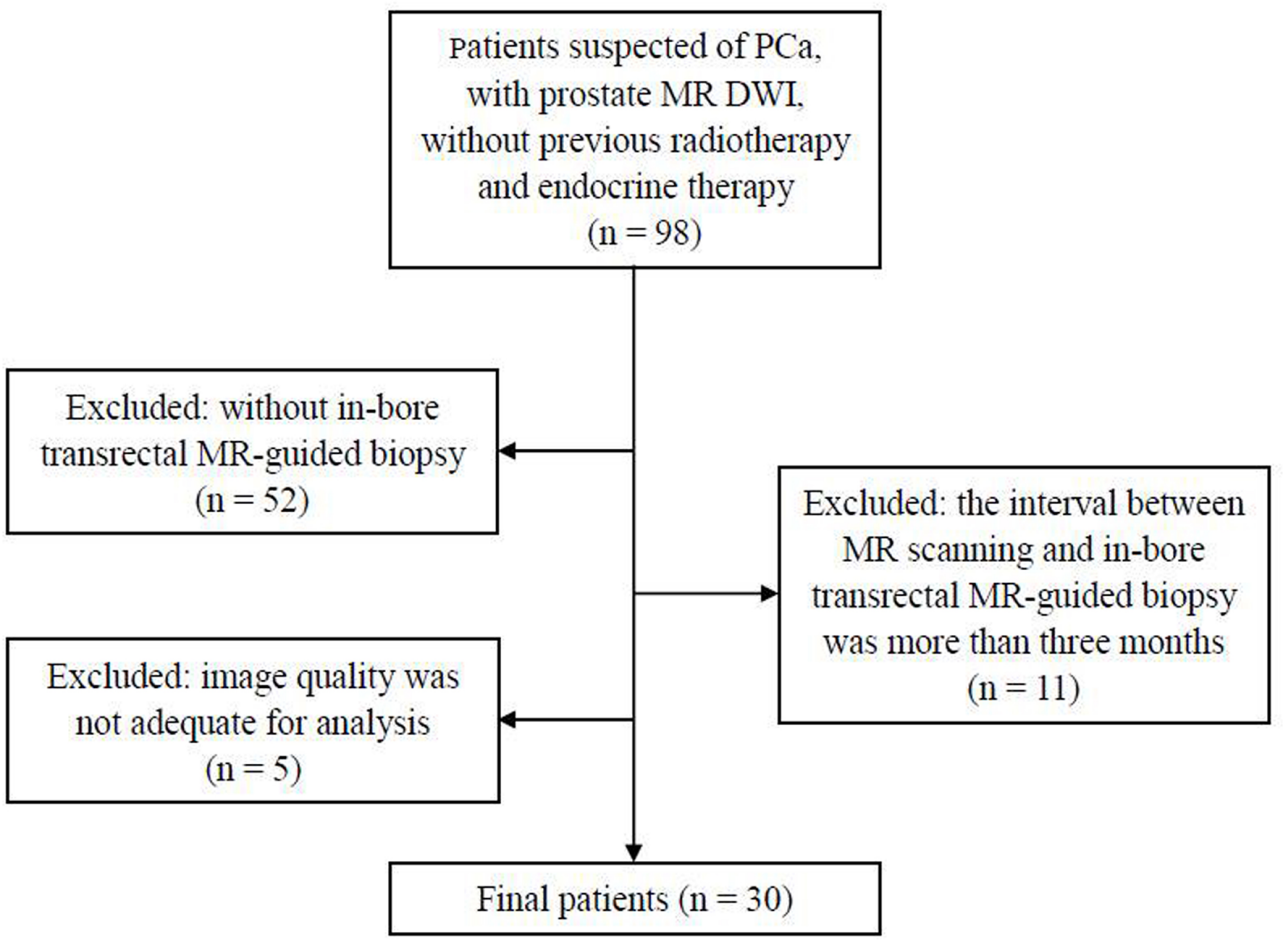
Flowchart shows the inclusion and exclusion criteria in the present study. PCa, prostate cancer; DWI, diffusion-weighted images.

The study was approved by our institutional review board and written informed consent was obtained from all the participants. A total of 98 patients suspected of PCa, without previous radiotherapy and endocrine therapy, were enrolled in this study from March 2017 to April 2019. The exclusion criteria were as follows: a) without in-bore transrectal MR-guided biopsy (*n* = 52), b) the interval between MR scanning and in-bore transrectal MR-guided biopsy was more than 3 months (*n* = 11), and c) image quality was not adequate for analysis (*n* = 5). Finally, 30 patients were included in this study (**Figure 1**). Forty lesions were collected (two lesions each from 10 patients and one lesion from the other 20 patients). The clinical and pathologic characteristics of the patients are summarized in **Table 1**.

**Table 1:** Summary of clinical and pathologic characteristics of patients.

	PCa	BPH/prostatitis	Statistics	*P*
Lesion number	20	20	–	–
Age (years)^a^	72 ± 8	69 ± 8	1.115	0.272
PSA (ng/ml)^a^	18.84 ± 17.85	6.07 ± 1.72	3.183	0.005**
Location				
Transition zone	12	16		
Peripheral zone	8	4		
Gleason score				
6 (3 + 3)	5			
7 (3 + 4)	7			
7 (4 + 3)	1			
8 (4 + 4)	3			
9 (5 + 4)	4			
PI-RADS v2.0				
3	5	19		
4	12	1		
5	3	0		

PCa, prostate cancer; BPH, benign prostatic hyperplasia; PSA, prostate-specific antigen.

a Data are means ± standard deviation.

**P < 0.01.

### MR Image Acquisition

MRI examinations were performed with a 3.0-T MR system (Achieva, Philips Medical Systems, Best, Netherlands). An eight-channel cardiac coil was used for signal reception. Images were acquired during free breathing. The scan protocol was as follows: standard axial, coronal and sagittal T2-weighted turbo spin echo (TSE) with fat saturation, axial T2-weighted TSE, axial T1-weighted TSE, and axial multiple *b*-values DWI. DWI was acquired using a single-shot echo-planar imaging with the following parameters: repetition time (TR)/echo time (TE) = 5,000/60 ms; slice thickness/gap = 4/1 mm; FOV = 220 × 200 mm; image matrix = 88 × 79; SENSE factor = 2; *b*-values (NEX) = 0 (2), 20 (2), 50 (2), 100 (2), 200 (2), 500 (3), 1,000 (4), 1,500 (6), 2,000 (6) s/mm²; gradient detection = 3. The DWI acquisition time was 7 min 57 s. The total scan time of prostate MRI was 21 min 46 s.

### In-Bore Transrectal MR-Guided Biopsy and Pathological Evaluation

MR-guided biopsy were conducted on a 3.0-T MRI scanner, with an MR-compatible biopsy device (Invivo, Schwerin, Germany) using an MR-guided biopsy system (DynaCAD Version 2.1.8). Suspicious lesions were identified as a PI-RADS v2.0 assessment category of ≥3 by two experienced radiologists (Chunmei Li and Min Chen, who had 10 and 20 years of experience in prostate MRI, respectively). The interventional biopsy was performed by another radiologist (Jingying Yu or Xiaotao Deng), and scanning operation was performed by one radiology technologist (Jintao Zhang). At least two specimens were obtained from each suspicious lesion.

The biopsy specimens were fixed in formalin and stained with hematoxylin–eosin before being analyzed by a pathologist (Wei Zhang). Each specimen was identified as PCa or benign prostatic hyperplasia (BPH)/prostatitis. Biopsy specimens with PCa were graded according to the 2014 International Society of Urological Pathology Gleason grading system. The lesion was grouped as PCa only if any one specimen was identified as PCa.

### Data Analysis

All DWI data were analyzed off-line with Matlab R2015b software (MathWorks Inc., Natick, MA, USA). Two different models, the IVIM-kurtosis model and MEM, were respectively used for postprocessing.

**Figure 2:**
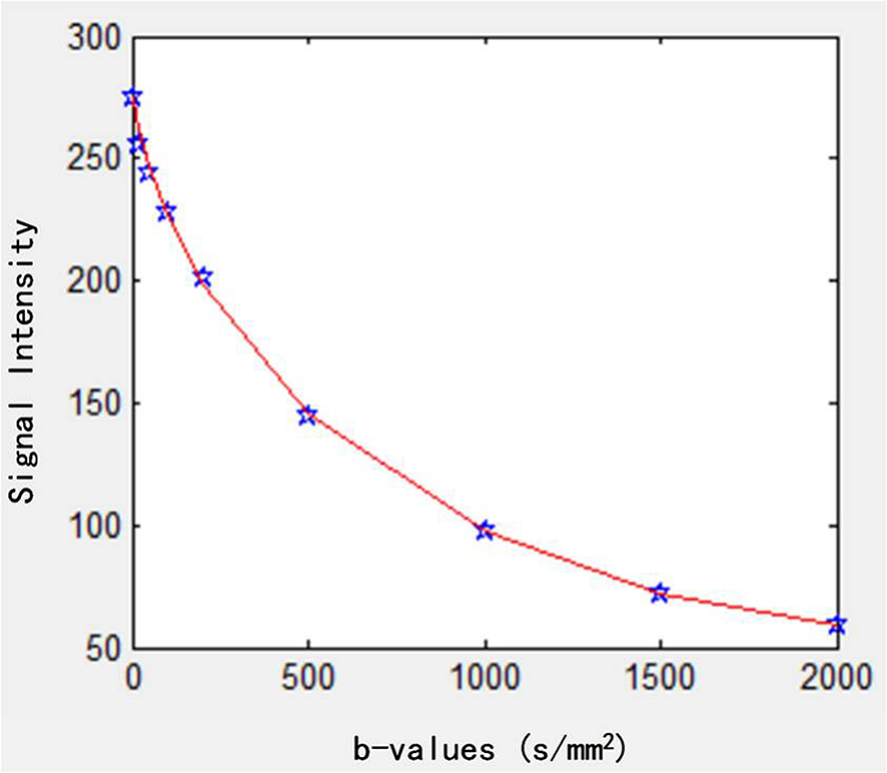
Fitting curve of the IVIM-kurtosis model. IVIM, intravoxel incoherent motion.

For the postprocessing of the IVIM-kurtosis model, the following equation was used (11):


$$S/S0=f\cdot \exp(-b\cdot D^{*})+(1-f)\cdot \exp[-b\cdot D+{\left(b\cdot D\right)}^{2}\cdot K/6]$$

where *S*(0) is the theoretical signal intensity for *b*-value of 0 s/mm^2^, *f* is the perfusion fraction, *b* is the *b*-value (*b*-values = 0, 20, 50, 100, 200, 500, 1,000, 1,500, 2,000 s/mm^2^), D* is the pseudodiffusion coefficient associated with the IVIM effect, *D* is the virtual diffusion coefficient, and *K* is the kurtosis parameter. The data fitting curve is shown in **Figure 2**.

The DWI was also analyzed using the MEM (23):


S(b)=S(0)exp (−b×ADC)

**Figure 3:**
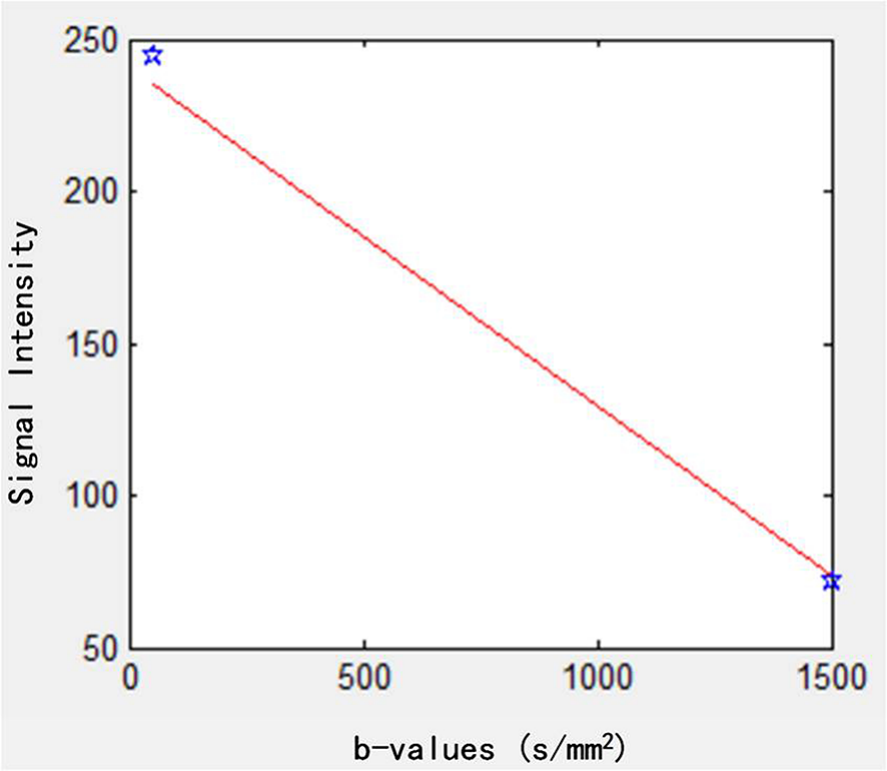
Fitting curve of MEM. MEM, monoexponential model.

ADC is the apparent diffusion coefficient and *b*-values were 50 and 1,500 s/mm^2^. The data fitting curve is shown in **Figure 3**.

Pixel-by-pixel maps of different parameters, including *D*, D*, *f*, *K*, and ADC, were automatically constructed from the proposed models. Regions of interest (ROIs) were performed by two radiologists (observer 1, Yuwei Jiang, and observer 2, Lu Yu, who have 5 and 6 years of experience in prostate MR imaging diagnosis, respectively, and were blinded to the final pathological results). ROIs were manually drawn on all suspicious lesions as described above, which underwent biopsy. The slice with the largest lesion area was used for ROI drawing. These two observers independently outlined the lesions. One observer (Lu Yu) underwent a second analysis 1 month later.

After ROI placement, histogram analysis of these parameters for each ROI was automatically generated (24). The mean, median, 10th percentile, 25th percentile, 75th percentile, 90th percentile, skewness, and kurtosis were acquired.

### Statistical Analysis

Statistical analysis was performed with SPSS 22.0 (IBM Corp., NY, USA) and MedCalc Statistical Software version 18.2.1 (MedCalc Software bvba, Ostend, Belgium). Intraclass correlation coefficient (ICC) was performed to assess intra- and interobserver repeatability of mean values and histogram parameters of the IVIM-Kurtosis model, and classified as poor (<0.40), fair (0.40–0.59), good (0.60–0.74), and excellent (0.75–1.00).

The normality of the data was tested using the Shapiro–Wilk test, and homoscedasticity was tested by Levene’s test first. Data were expressed as mean ± standard deviation (SD) or median (first quartile, third quartile) depending on whether following normal distribution. The parametric test (independent-sample *t*-test) would be applied to compare the difference of *D*, D*, *f*, *K*, and ADC between PCa and BPH/prostatitis when normality assumptions were satisfied; otherwise, the equivalent non-parametric test (Wilcoxon rank sum test) would be used.

The diagnostic performance of mean values and histogram parameters, which showed significant difference between PCa and BPH/prostatitis, was assessed by receiver operating characteristics (ROC) curves analysis. The area under the ROC curve (AUC) and 95% confidence interval (95% CI) were calculated. The sensitivity, specificity, and 95% CI were calculated with the maximum Youden index as the cutoff value. Spearman rank correlation analysis was used to evaluate the correlation of these parameters and Gleason score (GS) of PCa.

The diagnostic models of the mean values and the histogram parameters for the IVIM-kurtosis model and MEM were made by logistics regression. ROC curves analysis were used for the IVIM-kurtosis model and MEM. *Z* test was used to compare the differences between the ROCs of the IVIM-kurtosis model and MEM. *P*-values less than 0.05 were considered statistically significant.

## Results

### Intra- and Interobserver Agreement

Intra- and interobserver agreement of mean values and the histogram parameters of IVIM-Kurtosis model are shown in **Table 2**. Overall, the mean values and most histogram parameters showed excellent intraobserver and interobserver agreement. However, some poor ICCs were also shown, including the intraobserver agreement of D* (skewness) and *K* (25th).

**Table 2:** Intra- and interobserver agreement of mean values and histogram parameters for IVIM-Kurtosis model.

		Mean	10th	25th	50th	75th	90th	Skewness	Kurtosis
ICC of intraobserver agreement	*D*	0.988	0.219	0.966	0.991	0.993	0.985	0.921	0.820
	D*	0.985	0.924	0.925	0.973	0.986	0.984	0	0.805
	*f*	0.966	0.972	0.949	0.952	0.975	0.952	0.935	0.756
	*K*	0.960	0.863	0	0.836	0.933	0.991	0.669	0.314
ICC of interobserver agreement	*D*	0.986	0.196	0.959	0.979	0.984	0.979	0.886	0.779
	D*	0.910	0.068	0.862	0.760	0.923	0.915	0.846	0.785
	*f*	0.671	0.882	0.119	0.988	0.840	0.894	0.762	0.681
	*K*	0.951	0.763	0.847	0.942	0.925	0.893	0.516	0.582

### Histogram Analysis Between PCa and BPH/Prostatitis

The detailed information of *D*, D*, *f*, *K*, and ADC in PCa and BPH/prostatitis are demonstrated in **Table 3**.

**Table 3:** The mean values and histogram parameters for D (×10^−3^ mm^2^/s), D* (×10^−3^ mm^2^/s), *f*, *K*, and ADC (×10^−3^ mm^2^/s) in PCa and BPH/prostatitis.

Parameters	PCa (*n* = 20)	BPH/prostatitis (*n* = 20)	Independent-samples *t*-test	
			Statistics	*P*
*D* (×10^−3^ mm^2^/s)				
Mean	0.85 ± 0.24	1.34 ± 0.30	−5.682	<0.001^***^
10th	0.47 ± 0.17	0.70 ± 0.32	−2.869	0.007^**^
25th	0.66 ± 0.22	1.09 ± 0.31	−5.036	<0.001^***^
50th	0.85 ± 0.26	1.39 ± 0.32	−5.859	<0.001^***^
75th	1.05 ± 0.30	1.67 ± 0.37	−5.812	<0.001^***^
90th	1.22 ± 0.36	1.88 ± 0.46	−5.059	<0.001^***^
Skewness	−0.04 ± 0.53	−0.35 ± 0.45	3.276	0.002^**^
Kurtosis	3.00 ± 0.96	2.85 ± 0.82	0.507	0.615
D* (×10^−3^ mm^2^/s)				
Mean	0.742 ± 0.560	1.038 ± 0.964	−1.192	0.241
10th	0.028 ± 0.117	0.003 ± 0.003	0.952	0.347
25th	0.094 ± 0.277	0.062 ± 0.175	0.437	0.664
50th	0.379 ± 0.565	0.650 ± 1.165	−0.935	0.356
75th	1.087 ± 0.999	1.869 ± 1.753	−1.733	0.091
90th	1.909 ± 1.327	3.181 ± 2.050	−2.330	0.025^*^
Skewness	2.178 ± 1.379	1.444 ± 1.125	1.844	0.073
Kurtosis	9.134 ± 8.269	4.685 ± 4.443	2.077	0.045^*^
*f*				
Mean	0.229 ± 0.107	0.246 ± 0.091	−0.536	0.595
10th	0.021 ± 0.019	0.032 ± 0.013	−2.203	0.034^*^
25th	0.050 ± 0.025	0.062 ± 0.024	−1.478	0.148
50th	0.137 ± 0.166	0.139 ± 0.065	−0.045	0.965
75th	0.301 ± 0.219	0.368 ± 0.194	−1.032	0.308
90th	0.662 ± 0.258	0.685 ± 0.269	−0.267	0.791
Skewness	1.497 ± 0.692	1.220 ± 0.700	1.260	0.215
Kurtosis	4.638 ± 2.377	4.040 ± 4.002	0.575	0.569
*K*				
Mean	1.396 ± 0.654	0.949 ± 0.341	2.712	0.010^*^
10th	0.140 ± 0.247	0.227 ± 0.259	−1.090	0.282
25th	0.462 ± 0.411	0.568 ± 0.253	−0.982	0.334
50th	0.896 ± 0.624	0.755 ± 0.200	0.964	0.345
75th	1.628 ± 1.401	0.927 ± 0.349	2.172	0.041^*^
90th	3.510 ± 2.778	1.927 ± 1.151	2.354	0.027^*^
Skewness	1.849 ± 1.805	2.113 ± 1.672	−0.481	0.633
Kurtosis	10.150 ± 9.527	10.148 ± 6.397	0.001	1.000
ADC (×10^−3^ mm^2^/s)				
Mean	0.712 ± 0.150	1.066 ± 0.193	-6.495	<0.001^***^
10th	0.582 ± 0.134	0.898 ± 0.189	−6.095	<0.001^***^
25th	0.635 ± 0.129	0.973 ± 0.194	−6.482	<0.001^***^
50th	0.708 ± 0.152	1.066 ± 0.197	−6.423	<0.001^***^
75th	0.778 ± 0.170	1.156 ± 0.204	−6.366	<0.001^***^
90th	0.845 ± 0.198	1.240 ± 0.208	−6.149	<0.001^***^
Skewness	0.258 ± 0.269	0.116 ± 0.516	1.096	0.280
Kurtosis	2.701 ± 0.529	2.634 ± 1.064	0.255	0.800

PCa, prostate cancer; BPH, benign prostatic hyperplasia.

*Significantly different at P < 0.05.

**Significantly different at P < 0.01.

***Significantly different at P < 0.001.

For the IVIM-kurtosis model, *D* (mean, 10th, 25th, 50th, 75th, 90th), D* (90th), and *f* (10th) were significantly lower in PCa than in BPH/prostatitis. Meanwhile, *D* (skewness), D* (kurtosis), and *K* (mean, 75th, 90th) were significantly higher in PCa than in BPH/prostatitis.

For MEM, ADCs (mean, 10th, 25th, 50th, 75th, 90th) were significantly lower in PCa than in BPH/prostatitis.

**Figure 4:**
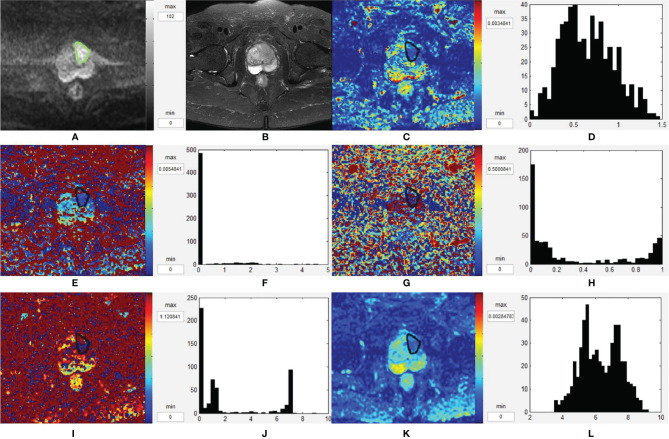
Representative images from a 76-year-old PCa patient with Gleason score of 3 + 4, as outlined on the images. **(A–L)** Axial DWI (*b* = 1,500 s/mm^2^), axial T2WI, *D* map, histogram of *D*, D* map, histogram of D*, *f* map, histogram of *f*, *K* map, histogram of *K*, ADC map, and histogram of ADC, respectively. PCa, prostate cancer; T2WI, T2-weighted images; DWI, diffusion-weighted images; ADC, apparent diffusion coefficient.

**Figure 5:**
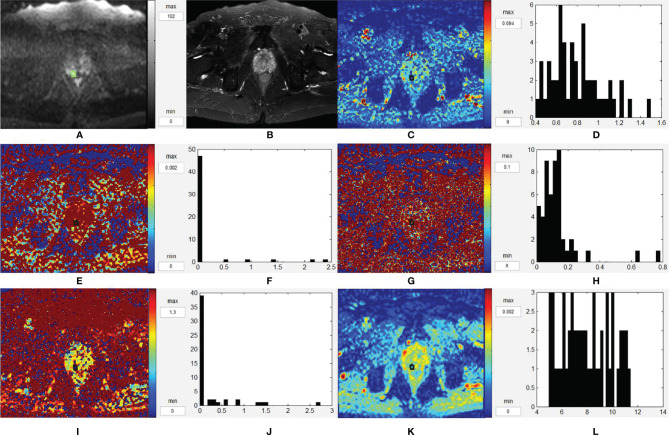
Representative images from a 63-year-old BPH/prostatitis patient, as outlined on the images. **(A–L)** Axial DWI (*b* = 1,500 s/mm^2^), axial T2WI, *D* map, histogram of *D*, D* map, histogram of D*, *f* map, histogram of *f*, *K* map, histogram of *K*, ADC map, and histogram of ADC, respectively. BPH, benign prostatic hyperplasia; T2WI, T2-weighted images; DWI, diffusion-weighted images; ADC, apparent diffusion coefficient.

**Figures 4** and **5** are two representative cases of PCa and BPH/prostatitis.

### ROC Analysis

The results of ROC analysis for mean values and histogram parameters, which showed significant difference between PCa and BPH/prostatitis, as well as the combination models of *D*, D*, *f*, and ADC, of the IVIM-kurtosis model and MEM, are shown in **Table 4**.

**Table 4:** The results of ROC analysis for mean values and histogram parameters.

Parameters	AUC (95% CI)	Sensitivity (%)	Specificity (%)	Youden index	Association criterion
*D* (×10^−3^ mm^2^/s)					
Mean	0.865 (0.720–0.952)	90.0 (68.3–98.8)	80.0 (56.3–94.3)	0.7000	≤1.190
10th	0.760 (0.599–0.881)	55.0 (31.5–76.9)	95.0 (75.1–99.9)	0.5000	≤0.400
25th	0.870 (0.726–0.955)	65.0 (40.8–84.6)	95.0 (75.1–99.9)	0.6000	≤0.704
50th	0.900 (0.763–0.972)	90.0 (68.3–98.8)	85.0 (62.1–96.8)	0.7500	≤1.212
75th	0.863 (0.717–0.951)	85.0 (62.1–96.8)	85.0 (62.1–96.8)	0.7000	≤1.333
90th	0.835 (0.684–0.933)	85.0 (62.1–96.8)	85.0 (62.1–96.8)	0.7000	≤1.510
Skewness	0.710 (0.545–0.842)	55.0 (31.5–76.9)	90.0 (68.3–98.8)	0.4500	>0.071
* D* model	0.927 (0.800–0.985)	85.0 (62.1–96.8)	90.0 (68.3–98.8)	0.7500	>0.480
D* (×10^−3^ mm^2^/s)					
90th	0.671 (0.505–0.811)	100.0 (83.2–100.0)	35.0 (15.4–59.2)	0.3500	≤4.272
Kurtosis	0.726 (0.562–0.855)	55.0 (31.5–76.9)	85.0 (62.1–96.8)	0.4000	>6.298
D* model	0.691 (0.526–0.827)	85.0 (62.1–96.8)	50.0 (27.2–72.8)	0.3500	>0.429
*f*					
10th	0.718 (0.553–0.848)	45.0 (23.1–68.5)	100.0 (83.2–100.0)	0.4500	≤0.014
*K*					
Mean	0.720 (0.556–0.850)	55.0 (31.5–76.9)	90.0 (68.3–98.8)	0.4500	>1.252
75th	0.867 (0.723–0.954)	90.0 (68.3–98.8)	75.0 (50.9–91.3)	0.6500	>0.946
90th	0.685 (0.519–0.822)	40.0 (19.1–63.9)	100.0 (83.2–100.0)	0.4000	>3.995
* K* model	0.780 (0.621–0.895)	70.0 (45.7–88.1)	95.0 (75.1–99.9)	0.6500	>0.543
IVIM-kurtosis model	1.000 (0.912–1.000)	100.0 (83.2–100.0)	100.0 (83.2–100.0)	1.0000	>0
ADC (×10^−3^ mm^2^/s)					
Mean	0.945 (0.824–0.992)	90.0 (68.3–98.8)	90.0 (68.3–98.8)	0.8000	≤0.845
10th	0.917 (0.786–0.981)	100.0 (83.2–100.0)	75.0 (50.9–91.3)	0.7500	≤0.742
25th	0.940 (0.817–0.990)	100.0 (83.2–100.0)	80.0 (56.3–94.3)	0.6000	≤0.704
50th	0.942 (0.820–0.991)	90.0 (68.3–98.8)	90.0 (68.3–98.8)	0.8000	≤0.849
75th	0.933 (0.806–0.987)	85.0 (62.1–96.8)	95.0 (75.1–99.9)	0.8000	≤0.890
90th	0.925 (0.796–0.984)	85.0 (62.1–96.8)	100.0 (83.2–100.0)	0.8500	≤0.938
ADC/MEM model	0.938 (0.813–0.989)	100.0 (83.2–100.0)	75.0 (50.9–91.3)	0.7500	≤0.289

**Figure 6:**
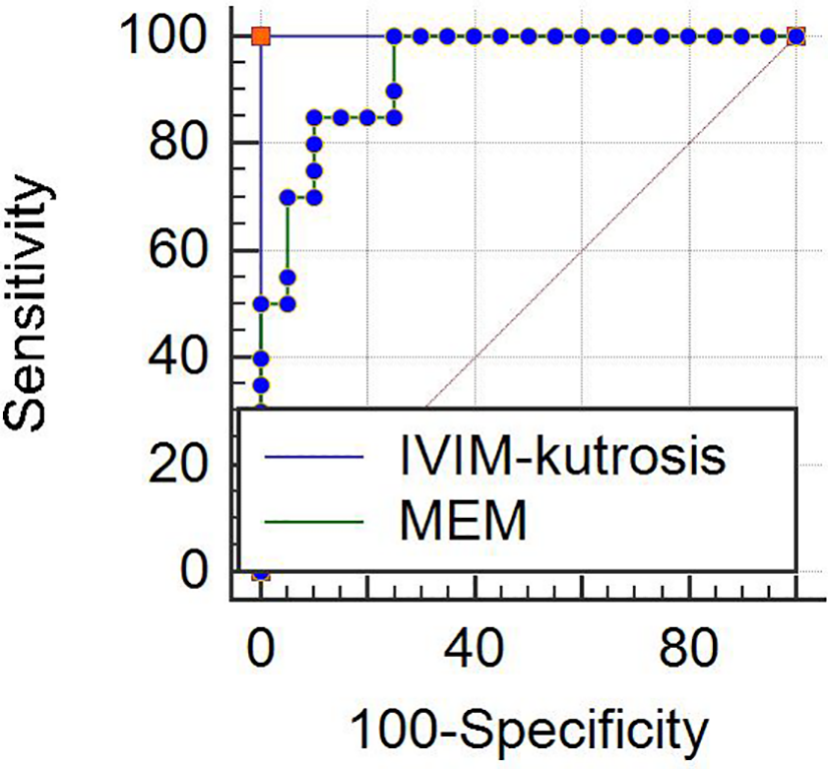
ROC curves for the IVIM-kurtosis model (AUC = 1.000) and MEM (AUC = 0.938). No significant difference was found between the two models (*z* = 1.761, *P* = 0.0783). Note: IVIM, intravoxel incoherent motion; MEM, monoexponential model.

The results of ROC analysis for IVIM-kurtosis model and MEM were showed in **Figure 6**. The AUC of IVIM-kurtosis model was higher than MEM (1.000 *vs.* 0.938), but no significant difference was found between these two models (*z* = 1.761, *P* = 0.0783).

### Correlations Between Parameters and GS

D (mean, 50th, 75th, 90th), D* (mean, 10th, 25th, 50th, 75th) and f (skewness, kurtosis) correlated negatively with GS, while D (kurtosis), D* (skewness, kurtosis), f (mean, 75th, 90th), K (mean, 75th, 90th) correlated positively with GS (**Table 5**). The other parameters derived from IVIM-kurtosis model, mean values and histogram parameters of ADC didn’t show correlations with GS.

**Table 5:** Spearman correlation of histogram IVIM-kurtosis and MEM parameters to tumor GS (with *P* < 0.05).

Parameters	*ρ*	*P*
*D* mean	−0.451	0.046^*^
*D* 50th	−0.518	0.019^*^
*D* 75th	−0.506	0.023^*^
*D* 90th	−0.448	0.048^*^
*D* kurtosis	0.510	0.021^*^
D* mean	−0.474	0.035^*^
D* 10th	−0.505	0.023^*^
D* 25th	−0.543	0.013^*^
D* 50th	−0.532	0.016^*^
D* 75th	−0.558	0.010^*^
D* skewness	0.501	0.024^*^
D* kurtosis	0.524	0.018^*^
*f* mean	0.703	0.001^**^
*f* 75th	0.679	0.001^**^
*f* 90th	0.760	<0.001^***^
*f* skewness	−0.685	0.001^**^
*f* kurtosis	−0.729	<0.001^***^
*K* mean	0.576	0.008^**^
*K* 75th	0.479	0.033^*^
*K* 90th	0.668	0.001^**^

IVIM, intravoxel incoherent motion; MEM, monoexponential model; GS, Gleason score.

*Significantly different at P < 0.05.

**Significantly different at P < 0.01.

***Significantly different at P < 0.001.

## Discussion

In this study, we firstly evaluated the repeatability of mean values and histogram parameters derived from the IVIM-kurtosis model. The results displayed that overall the repeatability is good. However, some parameters showed extremely low ICC, including the intraobserver agreement of D* (skewness) and *K* (25th). This may be due to the dominant heterogeneity of these parameters, which may cause match errors when the ROI selection was only a little bit mismatched. Besides, the ICC of histogram parameters seemed to be similar to the ICC of traditional mean value, which indicated that the histogram parameters may not have better repeatability than traditional mean value. In general, the IVIM-kurtosis model has good repeatability, but the reason for poor repeatability of some parameters still needs confirmation and further improvement.

The IVIM-kurtosis model allows the simultaneous measurement of perfusion and diffusion. It was reported that the IVIM-kurtosis model could be used to assess cerebral perfusion and diffusion heterogeneity in healthy adults and brain tumors (12, 25). The study of Lu et al. demonstrated that the IVIM-kurtosis model can address the underlying diffusion and perfusion behavior better than the IVIM and DKI models (26), and Fujima et al. found that the *D* and *K* values obtained by the hybrid IVIM-DKI model can be one of the diagnostic tools for the prediction of future distant metastasis in head and neck cancer patients (13). We are the first to evaluate the potential of the IVIM-kurtosis model in prostate cancer. In our study, we found that *D* (mean) significantly declined in PCa compared with that in BPH/prostatitis, which was consistent with previous literature (5, 27). This may be attributed to the presence of abundant fibrotic components and stromal fibroblasts or other microstructural differences in PCa. Similarly, *D* (10th, 25th, 50th, 75th, 90th, skewness) can be used for differentiating PCa from BPH/prostatitis, mostly with high sensitivity and specificity. For the histogram of *D* derived from the IVIM model, their efficiency in differentiating PCa from BPH/prostatitis is controversial (28, 29). For the histogram of *D* derived from the DKI model, our previous study showed that all the percentage of *D* can be used for the diagnosis of PCa (30), similar to the histogram of *D* derived from the IVIM-kurtosis model in this study. Thus, we infer that the histogram of *D* derived from the IVIM-kurtosis model may obtain more meaningful histogram parameters than the IVIM model and provide more information for the diagnosis of PCa.

D* and *f* reflect the perfusion of tumor. Agreements have not been achieved yet for their value in the diagnosis of PCa. Liu et al. found that PCa in central gland had lower D* than BPH and D* can improve the accuracy of PCa diagnosis (31), while Li et al. demonstrated that D* was higher in PCa than that in prostatitis and no significant difference existed between PCa and BPH. Moreover, the study of Shinmoto et al. showed no significant differences in PCa, BPH, and peripheral zone for D* (32). In this study, we did not further classify the lesions by the anatomical zones of the prostate, but the results indicated that D* (mean) could not differentiate PCa from BPH/prostatitis. Meanwhile, the results displayed that D* (90th) in PCa was significantly lower than that in BPH/prostatitis, while D* (kurtosis) in PCa was significantly higher than that in BPH/prostatitis, which is different from the results of Cui et al. (28). Feng et al. evaluated the effect of echo time value on IVIM quantification and showed that the accurate measurement of D* is still challengeable (33). D* is prone to errors, particularly when *f* gets very small (34). The different gradient directions and relatively isotropic diffusion in the prostate gland may also affect D*. The accurate evaluation of D* require further studies.

The value of *f* in PCa and non-cancerous, as well as their differences, was highly variable within previous studies. The *f* did not yield valuable information for the detection of PCa (10), while another study found *f* increased (35) or decreased in tumors (28, 31). In our study, the *f* (mean) did not show significant differences between PCa and BPH/prostatitis. Among the histogram parameters, only *f* (10th) had significantly lower value in PCa than that in BPH/prostatitis. Cui et al. found that the values of *f* (mean, 10th, 25th, 50th, 75th and 90th) were significantly lower in PCa than those in BPH (28), while Bao et al. found that histogram analysis could not differentiate PCa from BPH (29). The diversity on *f* may be due to that the perfusion fraction depended heavily on the *b*-values. Pang et al. (35) investigated the effect of *b*-values on perfusion fraction and found that when high *b*-values were used, the *f* became lower or indistinguishable from normal corresponding to prior reports. However, there is still limited consensus about the optimal *b*-value selection in prostate DWI until now. Another explanation is that separation of perfusion from diffusion requires high signal-to-noise ratios, and there are some technical challenges affecting *f* values, such as artifacts from other bulk flow phenomena. Besides, the different models used in previous studies and in this study may also contribute to the different results to some extent.

In theory, *K* may provide a more specific measurement of the complexity degree of microstructure of tissues. Suo et al. reported that *K* in malignant lesions was significantly higher than that in benign lesions (36). Moreover, Quentin et al. found that mean kurtosis and axial kurtosis derived from DKI could significantly differentiate PCa from prostatitis, the peripheral zone, or the central gland (37). However, Matthias et al. demonstrated no significant benefit of DKI for the detection and grading of PCa as compared with standard ADC determined from *b*-values of 0 and 800 s/mm^2^ (38). *K* (mean, 75th, 90th) showed significant differences between PCa and BPH/prostatitis in our study. This can be explained by the more complexity degree of microstructure in PCa than BPH/prostatitis. The diversity of *K* may also be ascribed to the different *b*-value selections and different models used, as we said in the discussion for *f*. Overall, the optimal *b*-value and model selection is crucial for the application of diffusion.

We also demonstrated the AUCs of histogram analysis of the IVIM-kurtosis model. Generally, the AUCs of *D* were higher than other parameters and the AUC of *D* parameters achieved 0.927, which was similar to the AUC of ADC parameters (0.938). It should be noticed that the AUC of the IVIM-kurtosis model reached 1.000. This too perfect result may be due to the following reasons. First, there may be a supplement between the parameters for the diagnosis of PCa. Second, though the AUCs were not high for most included parameters, some cutoff value may accidently exist, which could completely discriminate PCa from BPH/prostatitis, when constructing the combined diagnostic model. Actually, previous studies found that IVIM and DKI models were not superior to MEM in the diagnosis of PCa (8, 28, 29, 39); thus, researchers did not recommend IVIM and DKI models for clinical application at present. In this study, the AUC of the IVIM-kurtosis model was higher than MEM (1.000 *vs.* 0.938). Though no significant difference was found between them, there was a trend that the IVIM-kurtosis model showed better diagnostic value than MEM for the diagnosis of PCa. We should also notice another important point. Though the Youden index of the IVIM-kurtosis model was better than MEM, the Youden index of MEM parameters was generally better than IVIM-kurtosis parameters. This indicated that MEM parameters may be better than IVIM-kurtosis parameters in the situation of separate application, while MEM parameters may be inferior to IVIM-kurtosis parameters in the situation of comprehensive application. This also indicated that histogram analysis of the IVIM-kurtosis model may provide a better diagnosis of PCa than mean value only. Considering the longer scan time and postprocessing time for the IVIM-kurtosis model, whether the IVIM-kurtosis model should be used as a routine tool for the diagnosis of PCa in the clinic needs further confirmation.

The mean value of *D*, D*, *f*, *K*, and some of their histogram parameters had significant correlations with GS in this study. Moreover, the *ρ* values of multiple histogram parameters seemed to be higher than mean value, which indicated that histogram analysis may provide better grading of PCa. Most previous studies on the IVIM model showed that *D* value had good correlation with GS (5, 24, 29). Increased GS of PCa leads to increased cell density and decreased extracellular space, which may result in the decrease of *D* value. However, Pesapane et al. (27) found that *D* could not differentiate high-grade PCa from low- and intermediate-grade PCa. Moreover, the results of the study of Pesapane et al. indicated that D* and *f* did not make sense in the grade of PCa. On the other hand, Valerio et al. (4) and Cui et al. (28) found that some values of D* significantly correlated with GS. Our results were not exactly the same as previous studies mentioned above. The possible reasons are as follows. First, the number of patients included in this study was relatively small and the percentage of low-grade PCa was relatively low. Second, the different selection of *b*-values may influence the results. A very important part for DWI data acquisition is the choice of *b*-values, which control the degree of diffusion weighting. However, definite standards of prostate DWI data acquisition and postprocessing have not been established yet. According to the study of Merisaari et al., different optimal *b*-value distributions were found for different models and/or parameters (40). As no previous reports focused on the application of the IVIM-kurtosis model in PCa, we did not get experience from previous studies for the selection of *b*-values. Here, we chose *b*-value distribution under consideration of both models, rather than choosing optimal *b*-value distribution for either the IVIM or kurtosis model, for the model we used in this study combined IVIM and kurtosis together. Whether the selection of *b*-values in this study was suitable for the IVIM-kurtosis model still needs confirmation. Third, some parameters showed a large degree of variation. Thus, increasing sample number, optimizing the selection of *b*-values, and making parameters stable are important for the clinical application of the IVIM-kurtosis model in the future. As to *K*, our results showed that *K* (90th) significantly correlated with GS, which agreed with the results of Wang et al. (22). However, the efficiency of other *K* parameters seemed to be different. We infer that *K* can be used to evaluate the grading of PCa. The *K* parameters which had statistic differences were different in different studies, and this may be due to different selection of histogram parameters. It deserves further investigation for the optimal parameter selection for *K* in the future. In a word, histogram analysis of the IVIM-kurtosis model has potential value for the assessment of PCa grading, but thorough studies are needed for the acquisition of standard parameters and more reliable results.

We also evaluated the efficiency of ADC in the assessment of PCa grading. The association between ADC and GS varied in different studies (41). The mean value and histogram parameters did not show significant correlations with GS in this study, which was different from previous studies (29, 39, 42). One possible reason was considerable intrasubject heterogeneity pathologically (43). Another possible explanation to this could be technical factors including field strength and coil arrangement. In addition, very high *b*-value is helpful for prostate cancer detection, but its value for evaluating tumor risk is limited. The IVIM-kurtosis model seemed to be superior to MEM in the assessment of PCa grading, but this needs to be confirmed by further studies with a large sample, for such results may contribute to the small sample and fewer low-grade PCa in this study.

There were several limitations to our study. First, this study is limited by the small number of patients included. The number of subgroup of each GS group was even smaller, especially the low-grade PCa group, which may bring bias to the results. Besides, we did not differentiate GS 3 + 4 from 4 + 3 and GS 10 was not included. Second, the lesions were not further classified by the anatomical zones (peripheral zone and transition zone). BPH and prostatitis were analyzed together and healthy prostate tissues were not included because it is difficult to identify healthy prostate tissues in elderly male. Third, we used the in-bore MR-guided biopsy as the pathological reference. This can improve the match accuracy of pathology and MRI (44), while the high cost limited the number of included patients. More patients should be included in the future for a profound and detailed study. Fourth, the data were not validated by another cohort in this study. Further data collection is needed in the future.

In conclusion, IVIM-kurtosis quantitative parameter histogram analysis is a valuable tool for distinguishing PCa from BPH/prostatitis. There was a trend that the IVIM-kurtosis model showed better diagnostic value than MEM for the diagnosis of PCa. The IVIM-kurtosis model may also provide more information in predicting the GS of PCa compared with MEM.

## Data Availability Statement

The raw data supporting the conclusions of this article will be made available by the authors, without undue reservation.

## Ethics Statement

The studies involving human participants were reviewed and approved by Beijing Hospital. The patients/participants provided their written informed consent to participate in this study.

## Author Contributions

CL and LY conducted the MRI data processing and statistical analyses and drafted the initial manuscript. YJ, YC, YL, HH, ML, WZ, JZ, and CZ contributed to data collection and analyses. KS assisted with data analysis and interpretation. MC designed the study and assisted with data interpretation and manuscript review. All authors contributed to the article and approved the submitted version.

## Funding

This work was supported by grants from Beijing Hospital Nova Project (BJ-2016-037) and Beijing Hospital Clinical Research 121 Project (BJ-2018-090).

## Conflict of Interest

KS is an employee of Philips Healthcare.

The remaining authors declare that the research was conducted in the absence of any commercial or financial relationships that could be construed as a potential conflict of interest.

## Publisher’s Note

All claims expressed in this article are solely those of the authors and do not necessarily represent those of their affiliated organizations, or those of the publisher, the editors and the reviewers. Any product that may be evaluated in this article, or claim that may be made by its manufacturer, is not guaranteed or endorsed by the publisher.
